# New York Odontological Society

**Published:** 1877-03

**Authors:** 


### ARTICLE III.

### *New York Odontological Society.*

Regular meeting of the Society held at the residence of Dr. W. A. Bronson, No. 8 East Thirty-fourth Street, Tuesday evening, October 17th, 1876. Vice-President Dr. B. Lord in the chair.

Dr. B. Lord reported that he had attended the sixteenth annual session of the American Dental Association, held in Philadelphia the first week in August. The attendance was quite large, from one hundred and fifty to two hundred delegates being present; that while nothing new or of especial interest was advanced, the general results must be highly beneficial; social and fraternal relations were cultivated and strengthened, and the interchange of views, privately as well as in a public manner, must lead in many instances to a more sincere and earnest regard for one another.

*Incidents of Office Practice.*—Dr. Geo. Bernard. I have here a specimen of dilaceration,—a left centrrl incisor. When the child from whose mouth it was taken was six years old, she fell and fractured the tooth. The tooth was supposed to have been knocked out and lost, but in the course of a year there was an eruption of what the mother supposed to be a new tooth. Upon probing, it was found to be the root of the fractured tooth. The root was then extracted, and it came out with the crown of the tooth fused to the root, at right angles, as you see. As I believe the society is making a collection of specimens, I thought this might be of some value, and so I present it.

On motion, the specimen was accepted with the thanks of the society.

Dr. Henry N. Dodge exhibited an appliance for rotating incisor teeth.

Dr. A. H. Brockway exhibited a metal syringe, having a spiral spring so arranged as to make it self-charging; also an abscess syringe with glass barrel, devised by Dr. J. N. Farrar, constructed with a long and very delicate nozzle, the discharge being accomplished by turning a screw, forcing out a drop of medicine at a time.

Dr. W. H. Dwinelle. A short time since a gentleman presented himself for treatment at my hands who had met with quite a severe accident, by which his two central incisors were badly shattered, each being broken off diagonally across its surface, involving a loss of a considerable portion of substance, and causing great disfigurement of the mouth. The right one I found had not only its nerve exposed, but considerably more than one-half of it was broken off in a vertical direction. On its lingual surface the enamel was skived off above the gum. I found this tooth exceedingly loose. At first, I was inclined to attribute the looseness to the fact that the alveolar process was considerably broken up and comminuted. After removing the nerve I carefully examined the walls of the nerve-canal with a glass, prospecting at the same time with an instrument terminating with a delicate right-angle point; at the upper third of the root it dropped into a groove or crack, which extended entirely around the walls of the cavity; by illuminating the root of the tooth, I could distinctly see that it was fractured at this point. I did not want to sacrifice the tooth until I had made a further effort, to save it; so I resorted to the expedient of filling the nerve canal in such a manner that it would be, after I got through, riveted and cemented together—so to speak.

My method of filling roots is the old time-honored one, by carrying up small conical rolls of foil upon delicate Swedish broaches into the nerve-canal, consolidating the whole as I proceed with proper nerve-pluggers, and increasing the diameter of the rolls of gold to correspond with the shape of the canal. In this instance I resorted to the additional expedient of mixing up a small quantity of osteo-plastic of a thin consistency, and dipping these conical pieces of gold into it before putting them into the tooth, so as to induce a cementation against the walls of the canal, which being of unusual diameter favored my project, by securing a large and substantial pillar of gold within itself. After this treatment the tooth was decidedly firmer in its position, and with a few days' treatment it became so firmly established that I built up an entire crown upon it, using the pulp-cavity as a basis for building up a plug of gold that should be like a rivet. I also insured the success of the operation by locating gold screws at different points, one above the gum-line. When the operation was completed, the entire lingual surface which had been broken away was renewed in gold even considerably above the gum line.

The tooth on the left side, also, had its nerve exposed, so as to necessitate its removal. Its canal was filled in the usual manner and a gold crown built upon it.

The patient was discharged a few days ago, at which time I was satisfied the operation was a complete success; all tumefaction and inflammation had entirely subsided; the alveolus and gums had assumed a healthy and normal condition: the loosened tooth was apparently as firm as ever in its position, and I find myself asking the question, whether or no the fractured portions of the root may not ultimately unite by osseous deposit?

Dr. O. E. Hill. When I promised to occupy the time of this body for a portion of the evening I supposed I would have abundant opportunity to prepare a full and clear report of the cases which I intended to relate; but I in common with most of you, have had all my leisure time taken up in entertaining friends going to or coming from the Centennial. Therefore I shall have to rely upon the few notes taken down as the cases were being treated, and upon my appointment book, for the essential facts and dates of what I have to give you, and, as my friend Atkinson would say, trust to the angels for the rest.

Master Shuttleworth, aged nine, came to my notice about the middle of October, 1873. His mother stated that he had been sickly from his birth, and for a long time had been subject to fits. Of late the fits had increased so that he now had them almost daily. About eight months previous she noticed "matter" on his pillows every morning; the amount had gradually increased until now his pillows were soaked. Some four months later he had a very severe illness, in fact, was given up, and supposed to be dead; certainly at the time he had more the look and odor of the dead than the living,—with colorless face, bloodless body, imbecility of muscle, and lack of power of locomotion ; for he could walk but a few steps or stand but a few minutes. Locally, the gums were engorged, the processes somewhat exposed, and most of the teeth gone ; those remaining swimming in the pus that exuded from everywhere, above and below ; altogether, the little fellow was in a fearful condition.

On the 24th of October I made an examination, and found the bone on the left side of the lower jaw, from the symphysis to a little posterior of the sixth-year molar (which was very loose,) diseased ; just in front of this tooth the bone was exposed and black, and through an opening in it the crown of the second permanent bicuspid could be seen, black, and with little or no attachment. The temporary teeth were absent.

On the right side of the lower jaw, at a point corresponding to the position occupied by the temporary cuspid and first molar (the cuspid absent, molar present,) I found the bone diseased, anterior and posterior, involving the processes of these teeth, and extending down on to the body of the bone. At a corresponding point on the upper jaw, involving the same teeth (both present,) the diseased condition seemed identical. At a point to be occupied by the central incisors (temporary ones absent) there was a small opening in the gum, through which, on introducing a probe, I found a diseased condition of the bone, but not so extensive as at the other points. One would expect, on probing here, at the age of nine years, to find the permanent incisors somewhere, but I could not discover them.

In this case our first step must be to relieve the stomach of the large amount of pus that has been continually entering it, and in its stead administer that which will stimulate and assist in the processes of assimilation and nutrition, thereby causing the production of healthy tissue in lieu of the abortive material heretofore supplied.

First, then, we syringe the parts several times a day with tepid salt-water, not making it too strong at first, but gradually adding salt as the child will bear it without discomfort. Nourishing diet and constitutional treatment are of the utmost importance; in fact, his whole system is so involved, so low, that it will require the greatest care to give it a start towards recuperation. The child being fond of rare or even raw meat, the diet question is very agreeably settled.

The constitutional treatment will be cod-liver oil and iron, a teaspoonful of the former between meals, and ten drops of the tincture of chloride of iron after meals.

Our next object will be to hasten the separation of the dead and dying bone from the living by forcing a line of demarkation, where one is not already set up, by the application to the diseased parts of *sulphuric acid* of various strengths, as may be required. At this sitting, applied aromatic sulphuric acid to the left side of lower jaw. This was done every day to the 29th, thoroughly cleansing the parts, and applying sulphuric acid of various strengths, from the aromatic to equal parts of acid and water.

Oct. 29th.—Found the process on the lingual surface of the left side of lower jaw at the posterior point (said point being one fourth of an inch back of the sixth-year molar,) nearly detached; made the separation complete. From that point forward to where the second bicuspid would naturally be the necrosed bone had no attachment. Here made another separation, and removed the sequestrum. Also removed the transverse process, anterior and posterior, to the sixth-year molar. Applied aromatic sulphuric acid at all points; advised tepid salt-water baths and porter with the oil.

*Nov. 1st.*—As expected, the process on the buccal surface, corresponding to that removed at the last sitting, was nearly detached at the posterior point. In short, was in precisely the same condition, and treated in the same manner. This leaves the sixth-year molar entirely without support, except such as it may have from the soft tissues surrounding it. It seems to have no attachment. It certainly is exposed to the end of its roots, so that whatever attachment it does have is directly on the end. Applied aromatic sulphuric acid at all points.

*Nov. 3rd*—The soft tissues begin to show decided improvement, and the discharge of pus is much less. Up to this time have not been particular where the acid did touch, as it could not go amiss. Applied the acid now only at each particular point requiring it; this being as near the boundary of the diseased bone as possible. This is readily done by saturating a bit of lint with the acid and carrying it directly to the desired point, absorbing all moisture from the pocket previously. Have cotton ready to take up any overflow of acid. I say overflow, because it is desirable to have the pocket full.

*Nov 5th.*—Removed from the right side of upper jaw the first molar, cuspid, their entire sockets, together with process, anterior and posterior, for three-sixteenths of an inch. Filled the cavity with cotton saturated with equal parts of sulphuric acid and water, keeping it there for fifteen minutes.

*Nov. 8th.*—Patient constitutionally much improved, and at all points where bone has been removed a decided healthy action exists; the remaining necrosed bone looser· Acid treatment as before.

*Nov. 12th.*—Extracted the right lower first temporary molar. Found the necrosed bone in its vicinity free, and removed it; the sequestrum extending from the lateral to, and particularly involving the process of the second temporary molar, and extending down on the body of the bone, on its labial surface, in a scale-like form, to nearly its lower border. Filled the cavity with cotton saturated with equal parts of sulphuric acid and water. In fact, treated it the same as the corresponding parts in the upper jaw. As I to-day see the result of that treatment, namely, the bone nearly covered, and that which is in view smooth and in condition to receive its natural covering, I can but think it the best treatment possible.

To day the bone on the left side of the lower jaw, at the symphysis, inside and out, also on the labial surface, all the way along to the posterior point, seems to be detached; but on the lingual surface it is still attached at about the center of the lower border, being free at the ends. Applied sulphuric acid, one-half water, at the point of attachment.

*Nov.* 15th.—Found the sequestrum last described entirely detached, and removed it.

*Nov. 17th.*—The parts doing well, except at the symphysis; there the bone is rough. Applied sulphuric acid, one-half water, as in its present condition the advance of the soft tissues will not be kindly received.

At a point to be occupied by the right superior central incisor introduced a pledget of cotton in the opening in the gum, for the purpose of enlarging it.

*Nov. 19th.*—The bone at the symphysis is in good condition to receive the soft tissues, and they are already advancing upon it. Dressed with phenol sodique.

Removed the pledget of cotton from the upper gum, and extracted several spiculæ of bone, the larger being three-eighths of an inch long and one eighth wide. Dressed with aromatic sulphuric acid.

Necrosed bone all removed; soft tissues in good condition. *In every instance, when dead bone was removed, healthy granulations were found beneath it* Directed phenol sodique as a wash for the mouth.

*Nov. 24th.*—Nothing required; continued the oil, but changed iron to the syrup of iodide of iron.

The little fellow walked to the office, a distance of half a mile; has very little color yet, but is rapidly gaining strength.

The condition of the permanent teeth is as follows:

The incisors, cuspid, and first bicuspid on the left side of lower jaw in full view, held in position by the soft tissues only, all the bone having been removed from around them the sixth-year molar firmer, in fact, safe; permanent teeth in view at all points where bone has been removed,—that is, they have been uncovered, except the superior central incisors, which cannot be seen, nor can they be found with a probe.

Case of necrosis and wasting away of the processes caused by an abscessed superior left central incisor, the tooth having been filled over a dead pulp. Patient a young lady ; age, twenty-five.

April 17th, 1876, made an examination, and found the process on the labial surface, from the median line to the posterior side of the left cuspid, gone or necrosed ; the transverse processes between the centrals, central and lateral, lateral and cuspid gone ; the diseased condition extending beyond the end of the roots of these teeth involving the maxillary bone on its facial surface to above the base of he nose, leaving only the outer form or cortex of the anterior nasal spine intact ; the gums engorged and purple, and readily lifted up, so that the ends of the roots of the central and lateral incisors were in full view. At the end of the root of the cuspid is a piece of necrosed bone, about three-eighths of an inch square, extending upward and towards the nares, free at all points except at the point of attachment at the end of the root of the cuspid.

We have now this condition:

The bone, from the median line to and including that on the anterior side of the cuspid, all wasted away, leaving the central and lateral incisors with no attachment, except a very slight one on their posterior surfaces ; in fact the central can be extracted with the fingers, the pulp in it being dead ; the pulp in the lateral probably alive, the gum covering only about one-eighth of an inch of the root of the central and about one-half the length of the root of the lateral. The surface of diseased bone is on a line immediately posterior to the central and lateral, except directly at the end of the lateral, where the disease has progressed more rapidly, making a cavity not less than a quarter of an inch deep, and of about the same diameter. The bone dark and exceedingly rough.

The important question in this case is, can we cause a reproduction of the parts and retain the teeth? Although advised not to attempt it, we hold it better to try and fail than not to try at all. Most certainly it would be bad practice to extract the teeth now. Patient ordered.

Syrupi ferri iodidi, f ʒ iv;

Potassii iodidi, ʒ ii;

Aquæ, f ℥ i;

Syrupi, q. s. ft. f ℥ iii.

Cleansed the parts with tepid salt and water, and syringed with aromatic sulphuric acid. Following day same treatment. The next day, which was the 19th, stuffed the cavity with lint wet with sulphuric acid and water, one to four.

*April 20th.*—Finding the bone still dark and not perceptibly affected by the acid, applied equal parts of sulphuric acid and water. This treatment daily for three days had the desired effect of disintegrating or dissolving the surface of dead bone, so that it was easily removed. From this date the exposed surface of diseased bone was daily touched with the lotion of acid and water, until its whole surface was covered with healthy granulations; after which, from time to time, we syringed the parts with wine of opium or aromatic sulphuric acid, as seemed best. In the mean time we filled the pulp-cavity of the central incisor with cotton saturated with creasote, changing it every day or two.

About the middle of July the patient left for the country, the cavity being nearly filled up; the teeth much firmer, the gums healing nicely around them. Saw the case again about the 1st of October. It is a perfect success; the lost parts restored, the teeth firm, and the gums firmly attached to them.

My reason for trying to save the teeth was, that by their extraction at that time there must have been a permanent loss of structure amounting to a deformity. With the teeth to hold out the soft tissues, I might cause a reproduction and building up around them sufficient to make the deformity less, even if the teeth had to be extracted at a later period. Fortunately, the teeth are now quite as firm as the others.

Third case. A young lady, twenty years of age, had a superior lateral incisor filled about eight months ago. Some two months afterwards she complained that cold water caused pain in it. I said to her, "Try it awhile longer, and if it continues to trouble you, come in again." She did not follow my directions, and in the course of time went into the country. While there the pulp of the tooth became inflamed, died, and resulted in an abscess, which filled the entire roof of the mouth. When I saw it, September 15th, it was discharging freely around the two left bicuspids. The lateral incisor not loose, and no indication of pus ln its immediate vicinity. I opened into the pulp-chamber, but found no pus there. I then made a small incision well back in the roof of the mouth, and by inserting the point of a syringe in the opening, and holding cotton tight around it, I could force its contents through the abscess and out at the point of discharge, (the bicuspids,) thus having a clear channel to wash through.

On examination found the bone on the left side, from the center to within less than one quarter of an inch of the teeth, and extending back to the palate-bone, entirely denuded of its periosteum, its surface quite rough and disintegrated.

Syringed out well with tepid salt and water, then with aromatic sulphuric acid. Repeated this treatment the next day. After that used sulphuric acid and water, one to four, every other day for ten days, at the same time treating the root of the lateral incisor with creasote as usual in abscessed teeth. At the end of three weeks ceased the acid treatment, using wine of opium instead. At four weeks the case was discharged cured, the only evidence of disease being a slight thickening of the soft tissues in the roof of the mouth.

I might relate other cases, but the above are, I trust, sufficient to prove that we have in sulphuric acid an agent that makes conservative surgery, especially about the mouth and face, not only possible, but easy and sure, and one, if judiciously used, that will not affect injuriously either the soft tissues of living bone.

While on this subject I will add, that since 1871 it has been my practice to use sulphuric acid in many cases where I formerly employed chloride of zinc or creasote, and with far more speedy and satisfactory results. For instance, it is not unusual for us to find fistulous openings in the gums. These fistulæ, perhaps not always, but as a rule, are dependent on a diseased condition of the bone, and are quite formidable—that is, they are very annoying to the patient, still, not enough so as to cause them to submit to any operation that will give them pain; hence it has always been difficult for me to successfully treat them, but now by injecting aromatic sulphuric acid, perhaps twice, (sometimes more,) the cure is complete. Where a fistula is formed from an abscessed tooth, the acid wtll not only assist in subduing the abscess, but it will dissolve and smooth the ragged edges on the end of the root, thereby presenting a surface not entirely antagonistic to the advance of the soft tissues.

Dr. William Jarvie, Jr. I have been much interested in the recital of these cases, particularly as I have seen them from time to time all through their treatment. I saw the boy once or twice before the operation was commenced, and saw him almost continuously during the treatment. The two latter cases I have seen two or three times during the course of the treatment, and I merely desire to testify to the unexaggerated and very modest manner in which Dr. Hill has presented them.

Not a single feature of the cases has been overstated. I think on the contrary, that in speaking of the amount of removal bone he has underestimated it.

On two occasions, with a rule, I measured the sequestrum. In one instance, I found he had recorded it to be but half its actual size, Dr. Hill judging from the eye, while I took the exact measure.

I might say one or two things about the boy, if the Doctor were absent, which I think, redound very much to his credit. I will say, however, that the child is of very poor parentage,—in fact, they were so poor that they were not able to provide sufficient nourishment for him, and consequently all that the Doctor did in this case was without hope of fee or pecuniary reward.

There is another point omitted by him in the boy's case to which I wish to call attention, viz: the effect of the acid upon the teeth.

As he stated, the germs or crowns of two or three of the teeth, now fully erupted and in place, were exposed and in full view, bobbing up and down in the soft tissues as they were touched. The acid, from almost pure sulphuric down to the aromatic, was frequently in contact with them, and yet to-day, as I saw them, the teeth were perfect. Their enamel is not disintegrated in the least. Why is this? My theory is that the germs or partially developed teeth were covered with Nasmyth's membrane, and that the acid does not affect the soft tissues. You all know enamel coming in contact with slightly diluted sulphuric acid would be dissolved quite readily.

The child, at the time the case was first treated, looked as though he might die at any moment. His health now, however, is very much improved, and seems firmly established. In fact, he has stood the small-pox and the measles since. The only teeth missing are the first and second bicuspids on the left side of the lower jaw. There is no doubt whatever but that the first bicuspid is lost. I am not certain that the second bicuspid has erupted.

The last case spoken of by Dr. Hill I saw yesterday, and, as far as I could judge, everything is in a perfectly healthy condition.

Dr. W. H. Atkinson. I would like to emphasize the importance of our doing thoroughly anything which we attempt.

These cases are perfect proofs of what I have been saying to dentists for many years, as to the action of sulphuric acid upon the soft and hard tissues, and especially as to the electrical affinity which sulphuric acid holds to dead bone.

I think the delineation of these cases would pay us well for not only one night's listening, but for a whole winter's study. I congratulate Dr. Hill and the profession upon the success attendant upon this thorough and energetic mode of treatment. He has said several things gratuitously which I would like to take up and eliminate from the beauty and truth of the presentation of the case as a whole. One point I will allude to. He said the bicuspid had been developed without having any bone covering. If he had understood the law of development of the teeth better he would not have made that statement.

Dr. Hill. If I said that I did not intend it.

Dr. Atkinson. I have reason to know numerous instances where living bicuspids have been removed under such circumstances as that, thinking they were dead, when they were only attached to the soft tissues, and would sway about under the touch of the finger or instrument.

There has been a great deal brought before us to-night which it would be well to review, and sift that which is useful from that which is deleterious, but it is so late that I will not pursue the subject further.

Dr. W. H. Dwinelle. I feel that we have reason to congratulate ourselves in view of the exposition which has been made here to-night. In this connection I am reminded of a lawsuit, which is now pending, although it is pretty evident what the decision will be. It is a case in which an honest and well-intentioned gentleman of our profession is being prosecuted for paralyzing a lady's jaw by the use of the aromatic sulphuric acid of the druggists, and using it very moderately and carefully. The lady came to me to act as a witness in her behalf. Upon questioning her I found she was not paralyzed at all, but was merely after money. I declined to do as she requested. I was ready, however, in company with Dr. Atkinson, and one or two others, to give my testimony in behalf of the dentist.

The point I wish to make here is this: Dr. Hill has been using sulphuric acid of almost full strength, and using it properly and wisely, showing that while a dead part will be affected by sulphuric acid, judiciously applied, a living tissue may not be to its injury; showing, too, that the great principle of vitality which underlies the whole human economy will accommodate itself to the most extraordinary extremes—as we find manifested in many other directions. A vitalized body will endure cold down to eighty degrees below zero with impunity, and from some of our tests in the Turkish baths I have reason to believe that the human system is able to endure a temperature of two-hundred and seventy degrees of heat with perfect safety. Whereas, strike out the vitality from the individual, and in one instance he is frozen to an icicle, and in the other he is cooked so thoroughly that he might be served up as a dish fit for canibals.

I wish to relate a case in which, several years ago, I cautiously and tentatively used sulphuric acid, and with the most satisfactory results. The patient presented herself to me with all her lower teeth, from the second bicuspid on the right side to the second bicuspid on the left, inclusive, much diseased, the alveolar processes entirely necrosed in front, and the bone extensively affected. There was a discharge of pus from every quarter. The most of the roots of the teeth were visible, and were also necrosed—dead. The alveolus was dark brown and black. A prominent surgeon advised me to remove all the teeth, as he was apprehensive that otherwise they might cause the patient' death from pyæmia. I went to work, however, and removed all the dead bone, and commenced the use of sulphuric acid freely. I then resorted to amputation of the roots one after another. I amputated the lower third, the lower half, and even more of some of them. Of one eye-tooth, I amputated the lower two-thirds, and then cut it up obliquely to the margin of the gum.

I succeeded not only in checking all flow of pus but in reproducing all of the lost alveoli and gums, so that they covered the roots of the teeth as in their normal condition. It was a singular fact in the case, that the periosteum and alveoli of the teeth along their *lingual* wall were unaffected by the disease, although in front they were almost wholly destroyed.

The result was that the lady retained her teeth in perfect order until her death, which occurred a few weeks ago, ten years after the operation. Adjourned.—*Dental Cosmos.*

